# Lipid Profile and Cardiovascular Risk Monitoring in Patients on Clozapine – an Audit at a Community Mental Health Resource Centre in Scotland

**DOI:** 10.1192/bjo.2024.628

**Published:** 2024-08-01

**Authors:** Aamir Shahzad

**Affiliations:** NHS Greater Glasgow and Clyde, Glasgow, United Kingdom

## Abstract

**Aims:**

Individuals with severe mental illnesses are at an increased risk of morbidity and mortality from cardiovascular diseases compared with the general population. Dyslipidaemia is a well-established contributor to CVD risk, alongside factors such as obesity, hypertension, smoking, diabetes, and a sedentary lifestyle. Many patients with severe mental illnesses often exhibit a combination of these risk factors. Notably, second-generation antipsychotics, particularly clozapine, are associated with a significant risk to elevate lipid levels. However, dyslipidaemia is a treatable condition, and various interventions are available to decrease the risk, ultimately reducing the associated morbidity and mortality. Therefore, NICE guidelines recommend monitoring of lipid profile initially at baseline, 3 months and then annually and cardiovascular risk assessment by validated tools like QRISK3 or Assign Score (validated tool used in Scotland).

The first aim of this audit was to see if a lipid profile had been done within the past 12 months in patients on clozapine treatments and second aim was to see if cardiovascular risk had been assessed using a validated tool i.e. Assign Score and lastly to check if lipid results and Assign Score had been communicated to the General Practitioner.

**Methods:**

The audit included 40 patients receiving clozapine treatment under the care of this local CMHT. We excluded 13 patients who were already on statin medication, those newly initiated on clozapine within the last three months or those who were aged below 30 years or above 74 years. The data collection spans from October 2022 to October 2023. Our analysis focused on bloods results in the last 12 months. After that, we searched for the cardiovascular risk assessment in last 12 months of patients’ electronic notes. Additionally, a comprehensive review of all communication records with General Practitioners was undertaken.

**Results:**

Lipid profile testing was done in 22 of 27 (81.1%) of the audited patients, revealing that a significant proportion, 59.9% (13 of 22), exhibited elevated total cholesterol levels exceeding 5mmol/L. However, the assessment of cardiovascular risk within the specified timeframe was notably low, with only 1 of 27 (3.70%) of the audited patients undergoing this evaluation. Furthermore, communication with General Practitioners (GPs) regarding lipid profiles was observed in a mere 4 of 22 (18.18%) of cases where such testing was conducted.

**Conclusion:**

The clinical audit showed a good level of compliance with lipid profile monitoring; however, notable deficiencies were noted in the assessment of cardiovascular risk and communication with GPs. These findings emphasized the need to enhance our compliance with protocols for a more comprehensive approach to safeguard the cardiovascular health of patients receiving clozapine. As a result, we have proposed improvement strategy at our local CMHT meeting involving the implementation of a structured process, wherein the clozapine clinic nurse initiates an electronic task for the relevant medic to review the results. The medic is then tasked with calculating the cardiovascular risk and communicating both lipid results and the risk assessment to the GP, ensuring their inclusion in the annual review correspondence and subsequent management. A repeat audit will be done after 12 months.

